# BMP9 exhibits dual and coupled roles in inducing osteogenic and angiogenic differentiation of mesenchymal stem cells

**DOI:** 10.1042/BSR20201262

**Published:** 2020-06-15

**Authors:** Haozhuo Xiao, Xiaoyu Wang, Claire Wang, Guangming Dai, Zhenglin Zhu, Shengqiang Gao, Baicheng He, Junyi Liao, Wei Huang

**Affiliations:** 1 Department of Orthopaedic Surgery, The First Affiliated Hospital of Chongqing Medical University, Chongqing 400016, China; 2 Department of Computational and Applied Mathematics, Rice University, Houston, TX 77005, U.S.A.; 3 Key Laboratory for Biochemistry and Molecular Pharmacology of Chongqing, Chongqing Medical University, Chongqing 400016, China

**Keywords:** Angiogenic differentiation, BMP9, MSCs, Osteogenesis-angiogenesis coupling, Osteogenic differentiation

## Abstract

Bone morphogenetic protein (BMP) 9 (BMP9) is one of most potent BMPs in inducing osteogenic differentiation of mesenchymal stem cells (MSCs). Recently, evidence has shown that osteogenesis and angiogenesis are coupled, however, it is unclear whether BMP9 induces MSC differentiation into endothelial-like cells and further promotes blood vessel formation. In the present study, we explored the potential of BMP9-induced angiogenic differentiation of MSCs, and the relationship between BMP9-induced osteogenic and angiogenic differentiation of MSCs. Osteogenic activities and angiogenic differentiation markers were analyzed at mRNA and protein levels. *In vivo* osteogenic and angiogenic differentiation of MSCs were tested by the ectopic bone formation model. We identified that adenoviral vectors effectively transduced in immortalized mouse embryonic fibroblasts (iMEFs) and expressed BMP9 with high efficiency. We found that BMP9 induces early and late osteogenic differentiation, and it up-regulated osteogenic marker expression in MSCs. Meanwhile, BMP9 induces angiogenic differentiation of MSCs via the expression of vascular endothelial growth factor a (VEGFa) and CD31 at both mRNA and protein levels. CD31-positive cells were also increased with the stimulation of BMP9. The ectopic bone formation tests found that BMP9-induced trabecular bone formation was coupled with the expression of blood vessel formation markers and sinusoid capillary formation. These findings suggest that BMP9 exhibits dual and coupled roles in inducing osteogenic and angiogenic differentiation of MSCs.

## Introduction

Mesenchymal stem cells (MSCs) are multipotent cells that can undergo self-renewal and hold the potential to differentiate into osteoblastic, myogenic, chondrogenic, and adipogenic lineages [1–3]. Owing to its ease of isolation, abundance in source, and persistent expression of exogenous genes, MSCs have been known as decent seed cells for regenerative medicine, including bone tissue engineering [4–7].

Bone morphogenetic proteins (BMPs) belong to the transforming growth factor β (TGF-β) superfamily and function as regulators in skeletal development, bone formation, and osteogenic differentiation of stem cell [8–10]. Through an integrated identification of the 14 types of BMPs in inducing osteogenic differentiation of MSCs, we characterized that BMP9 was one of the most osteogenic BMPs in inducing osteogenic differentiation of MSCs [11–14]. In the past decade, underlying mechanisms in BMP9-induced osteogenic differentiation of MSCs have been explored extensively [15], including Notch signaling [16–18], TGF-β signaling [19], epidermal growth factor (EGF) [20], classic Wnt signaling [21–23], Nell 1 signaling [24], long noncoding RNA H19 (lncRNA H19) [25], insulin-like growth factors (IGFs) [26], all of which were reported to participate in BMP9-induced osteogenic differentiation. Nonetheless, increasing evidence has shown that osteogenesis can not exist effectively without angiogenesis during the processes of bone development and bone formation [27–29], so it is indispensable to clarify underlying mechanisms of BMP9-induced osteogenesis-angiogenesis coupling of MSCs for the construction of BMP9-based bone tissue engineering.

In previous study, we identified that hypoxia-inducible factor 1α (Hif 1α)-mediated angiogenic signaling was essential for BMP9-induced osteogenesis of MSCs [30]. At the same time, we identified that Notch1 signaling potentiated BMP9-induced osteogenic differentiation of MSCs by potentiating the osteogenesis-angiogenesis coupling [16]. In addition, we clarified that Schnurri-3 simultaneously regulates BMP9-induced osteogenic differentiation and blood vessel formation in human amniotic MSCs [31]. The research indicated that angiogenic signaling participated in BMP9-induced bone formation of MSCs. However, it is still unclear whether BMP9 induces MSCs to differentiate into endothelial-like cells and further promotes blood vessel formation in BMP9-induced bone formation.

In the present study, we confirm that BMP9 exhibits dual roles in inducing osteogenic and angiogenic differentiation of MSCs, and at the same time, we identify that BMP9-mediated osteogenic differentiation was coupled with the expression of blood vessel formation markers and sinusoid capillary formation. These findings contribute to a new understanding of BMP9-mediated osteogenic and angiogenic differentiation, which is beneficial for the construction of BMP9-mediated bone tissue engineering.

## Materials and methods

### Cell culture and chemicals

The HEK 293 cell line was purchased from ATCC (Manassas, VA). The MSC cell line, immortalized mouse embryonic fibroblasts (iMEFs), was characterized as before [25,32,33]. Cells were maintained in complete Dulbecco’s modified Eagle’s medium (DMEM, HyClone, China), which contained 10% fetal bovine serum (FBS, Gibco, Australia), 100 U/ml penicillin, and 100 mg/ml streptomycin and were incubated in a carbon dioxide (CO_2_) incubator at 37°C in 5%. Unless indicated, all chemicals were purchased from Corning corporation or Sigma–Aldrich.

### Recombinant adenoviruses mediated expressing of BMP9

Recombinant adenoviruses were generated and amplified using AdEasy technologies as described previously, and the GFP vector was used as control [34–36]. In brief, the coding regions of GFP and human BMP9 were amplified with polymerase chain reaction (PCR), and subcloned to adenoviral shuttle vectors. Then the vectors were used to produce recombinant adenoviruses in HEK 293 cells. The newly generated adenoviruses were named as AdGFP and AdBMP9.

### RNA isolation and quantitative reverse transcription PCR

Total RNA was isolated with TRIzol regent (Invitrogen, CA, U.S.A.) following manufacturer’s protocol, then reverse transcription reactions were carried out with the use of PrimeScript RT reagent kit (Takara, Dalian, China). The quantitative PCR analysis was done using the CFX96 Real time PCR Detection System (Bio-Rad, CA, U.S.A.) with the SYBR Premix ExTaqII kit (Takara, Dalian, China) according to the manufacturer’s instructions. The steps for real-time PCR were as follows: 95°C for 30 s, 95°C for 5 s, and 60°C for 5 s, repeating 40 cycles. All values were normalized to GAPDH expression with the 2^−△△*C*_t_^ method. The primer sequences used for qPCR analysis are listed in Table 1.

**Table 1:** Primer oligonucleotide sequences used for PCR

Gene	Forward primer (5′–3′)	Reverse primer (5′–3′)
human BMP9	CCTGGGCACAACAAGGAC	CCTTCCCTGGCAGTTGAG
mouse Runx2	CCGGTCTCCTTCCAGGAT	GGGAACTGCTGTGGCTTC
mouse Osterix	GGGAGCAGAGTGCCAAGA	TACTCCTGGCGCATAGGG
mouse BSP	AGGGAACTGACCAGTGTTGG	ACTCAACGGTGCTGCTTTTT
mouse OPN	CCTCCCGGTGAAAGTGAC	CTGTGGCGCAAGGAGATT
mouse VEGF-a	TGACGGACAGACAGACAGACAC	ACGGCTACTACGGAGCGAGAAG
mouse CD31	TGCTCTCGAAGCCCAGTATT	TGTGAATGTTGCTGGGTCAT
mouse GAPDH	CTACACTGAGGACCAGGTTGTCT	TTGTCATACCAGGAAATGAGCTT

### Alkaline phosphatase activity assay

The alkaline phosphatase (ALP) activities were examined using the modified Great Escape SEAP chemiluminescence assay (BD Clontech) and/or histochemical staining, as described before [16,37]. For ALP histochemical staining, iMEF cells were induced for osteogenic differentiation using AdBMP9, and AdGFP was used as control. At indicated time points, cells were first fixed with 0.05% glutaraldehyde at room temperature for 10 min, then washed with distilled water, and finally subjected to histochemical staining with a mixture containing 0.1 mg/ml of napthol AS-MX phosphate and 0.6 mg/ml of Fast Blue BB salt and stored in the dark for 10 min. Histochemical staining was recorded with the use of bright light microscopy.

For the chemiluminescence assays, iMEFs were first lysed by cell lysis reagent (Promega, U.S.A.), and then 5 μl of the cell lysis liquid, 5 μl substrate (BD Clontech), and 15 μl Lupo buffer were fully mixed and stored in the dark at room temperature for 20 min. ALP activities were normalized by total cellular protein concentrations in each sample. Each assay condition was performed in triplicate, and the results were repeated in three independent experiments.

### Matrix mineralization assay

Alizarin Red S staining was used to detect the matrix mineralization of iMEFs. Cells were seeded in 24-well plates and infected with adenovirus vectors. Infected cells were cultured in a medium containing 50 mg/ml ascorbic acid and 10 mM β-glycerophosphate. On days 7 and 9, mineralized matrix nodules were stained for calcium precipitation by Alizarin Red S staining, as described previously [12,13,18,37]. In brief, cells were first fixed with 4% paraformaldehyde for 30 min, then washed with Phosphate Buffer Solution (PBS, pH = 4.2) and incubated with 2% Alizarin Red S solution for 30 min, followed by extensive washing with PBS. The staining of calcium mineral deposits was detected by bright light microscopy.

### Western blot analysis

The cell lysates were prepared using cell lysis buffer containing a protease inhibitor phenylmethanesulfonyl fluoride (PMSF, Beyotime, Shanghai, China). Protein (60 μg) was loaded on to 10% sodium dodecyl sulfate/polyacrylamide gel electrophoresis (SDS/PAGE) gel. After electrophoresis, proteins were transferred to a polyvinylidene fluoride (PVDF) membrane. The PVDF membranes were incubated overnight at 4°C with primary antibodies against vascular endothelial growth factor a (VEGFa; Abcam, Cambridge, U.S.A.), CD31 (Abcam, Cambridge, U.S.A.), and GAPDH (Bioworld Technology, MN, U.S.A.) at a dilution of 1:500 or 1:1000. Then, the PVDF membranes were subjected to incubation with a secondary antibody conjugated with horseradish peroxidase (HRP). Immune-reactive signals were determined by the electrochemical luminescence (ECL) kit (Millipore, MA, U.S.A.).

### Flow cytometry analysis

The cells were inducted with BMP9, and at scheduled time points, the cells were harvested and fixed in 4% paraformaldehyde for 10 min. Then, the cells were permeabilized (0.25% Triton X-100 on ice for 15 min), washed, and incubated with primary CD31 antibodies at 4°C for 1 h, followed by incubation with the secondary antibody at 4°C for 30 min. Finally, cells were incubated in buffer containing 50 mg/ml propidium iodide and 100 mg/ml RNase A for 30 min, and 1 × 10^5^ cells per sample were analyzed.

### Subcutaneous MSCs implantation and ectopic bone formation

The iMEF cells were infected with AdGFP or AdBMP9. One day after infection, cells were harvested and resuspended in PBS containing 300 U/ml penicillin and 300 mg/ml streptomycin. Then, approximately 80 μl of PBS (containing 5 × 10^6^ cells) were injected subcutaneously into the flanks of athymic nude (nu/nu) mice (4-week-old males, Experimental Animal Center, Chongqing Medical University, Chongqing, China). The ectopic masses were retrieved for next histological and micro-CT evaluations at 3, 4, and 5 weeks. The volumes of the ectopic bone masses were calculated using vernier calipers.

### Micro-computed tomographic imaging analysis

The specimens were imaged with micro-computed tomography (μCT, VivaCT 40, SCANCO Medical AG, Switzerland). The μCT system contains a tungsten target X-ray tube, with an X-ray source of 70 kVp and 114 μA. The image data analysis and three-dimensional (3D) reconstruction were performed using the scanner software (μCT 516.1). Bone volume (BV)/total volume (TV) (BV/TV) (%) were measured for each sample.

### Hematoxylin and Eosin staining

The samples were fixed with 10% formalin, decalcified with ethylene diamine tetraacetic acid (EDTA), and then embedded in paraffin. Serial sections with a thickness at 5 μm were carried out, and then mounted on to treated slides. The sections were deparaffinized and rehydrated in a graduated fashion with alcohols, and subjected to Hematoxylin and Eosin (H&E) staining as described previously [16,25,37].

### Immunohistochemical staining

The deparaffinized and rehydrated sections were first subjected to antigen retrieval. After being washed with PBS, the sections were blocked with 5% donkey serum and then incubated with CD31 (Abcam, Cambridge, U.S.A., 1:100 dilution) or Kinase Insert Domain Receptor (KDR) primary antibody (Abcam, Cambridge, U.S.A., 1:100 dilution) at 4°C overnight. After being sufficiently washed, the sections were incubated with biotin-labeled secondary antibody for 30 min, followed by incubation with streptavidin–HRP conjugate at room temperature for 20 min. The presence of the target proteins were detected by diaminobenzidine (DAB) staining and examined under the microscope (Olympus, Japan). Staining without the primary antibody was utilized as negative control. Semi-quantitative analysis of KDR-positive cells and CD31-positive cells were performed by ImageJ software.

### Statistical analysis

Quantitative data were expressed as mean ± standard deviation (SD). Statistical analysis was carried out using the SPSS 19.0 statistical software (IBM, NY, U.S.A.). One-way analysis of variance (ANOVA) and Student’s *t* test were used to determine the statistical significance with a cutoff of *P*<0.05.

## Results

### Adenovirus-mediated gene transduction and expression of BMP9

**Figure 1:**
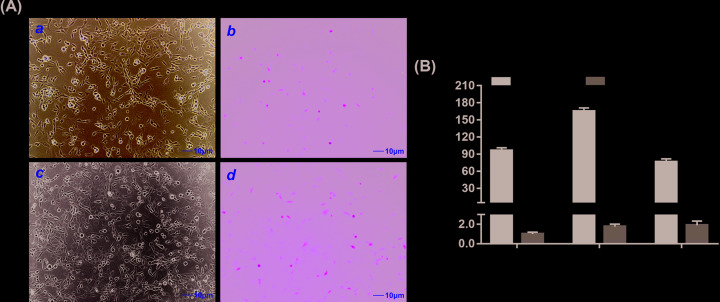
Adenovirus-mediated gene transduction and BMP9 expression (**A**) Exponentially growing iMEFs were infected with AdBMP9 or AdGFP, 24 h after the infection, bright light (**a,c**) and fluorescence microscope (**b,d**) examination showed the transduction efficiency of recombinant adenoviruses in monolayer culture. Scale bar = 10 μm. (**B**) Recombinant adenovirus-mediated overexpression of BMP9 were evaluated by RT-qPCR. At indicated time points, total RNA of each group was isolated and subjected to RT-qPCR analysis. All samples were normalized with the reference gene GAPDH. Relative expression was calculated by dividing the relative expression values (i.e., gene/GAPDH) in AdBMP9 group with that from the AdGFP group. Each assay condition was done in triplicate. ‘**’*P*<0.01, AdBMP9 group vs. AdGFP group.

The iMEF cells were cultured in a monolayer culture, and 12 h after the passage, recombinant adenoviruses expression GFP or BMP9 were added in the medium. As shown in Figure 1A, the green fluorescence indicated that adenoviruses could successfully infect iMEF cells 24 h after the transduction. The expression levels of BMP9 were determined by quantitative reverse transcription PCR (RT-qPCR) 3, 5, and 7 days after the transduction. Compared with AdGFP group, BMP9 was up-regulated dramatically by AdBMP9 on day 3, reached peak level on day 5, and then decreased appropriately on day 7 (Figure 1B). These results suggested that adenovirus-mediated overexpression of BMP9 was effective and sustained over 7 days.

### BMP9-induced osteogenic differentiation of MSCs

**Figure 2:**
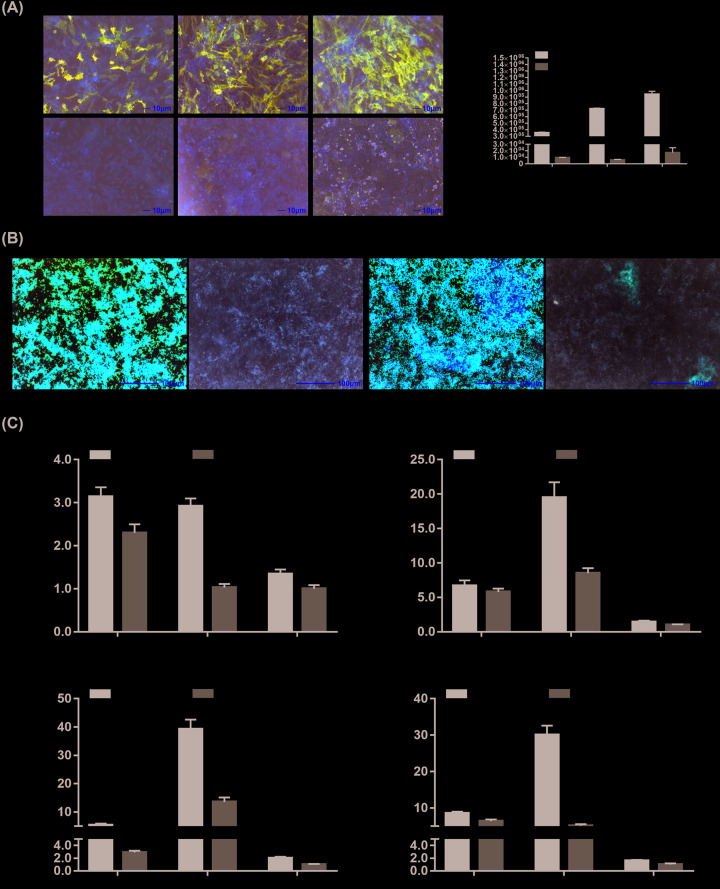
BMP9-induced osteogenic differentiation of MSCs *in vitro* (**A**) BMP9-induced ALP activities in MSCs. Subconfluent iMEF cells were infected with AdBMP9 or AdGFP. At the indicated time points, the infected cells were subjected to ALP activity assays by either histochemical staining (**a**) or quantitative the bioluminescence assay (**b**). Each assay conditions were done in triplicate. Representative staining is shown, scale bar = 10 μm. ‘*’*P*<0.05, ‘**’*P*<0.01, AdBMP9 group vs. AdGFP group. (**B**) BMP9-induced matrix mineralization in MSCs. Subconfluent iMEF cells were infected with AdBMP9 or AdGFP. At the indicated time points, the cells were subjected to Alizarin Red S staining. Each assay conditions were done in triplicate. Representative staining is shown, scale bar = 100 μm. (**C**) BMP9-induced osteogenic differentiation markers expression in MSCs. Subconfluent iMEF cells were infected with AdBMP9 or AdGFP. At the indicated time points, total RNA of each group was isolated and subjected to RT-qPCR analysis. Key osteogenic transcription factors Runx2 (**a**), Osterix (**b**), and late osteogenic differentiation marker BSP (**c**), OPN (**d**) were determined. All samples were normalized with the reference gene GAPDH. Relative expression was calculated by dividing the relative expression values (i.e., gene/GAPDH) in AdBMP9 group with that from the AdGFP group. Each assay condition was done in triplicate. ‘*’*P*<0.05, ‘**’*P*<0.01, AdBMP9 group vs. AdGFP group.

To confirm the osteogenic differentiation potential of iMEF cells by the inducing of BMP9, ALP assays, Alizarin Red S staining, and osteogenic differentiate markers were determined. As an early osteogenic differentiation marker, ALP activities were evaluated by ALP staining and ALP quantitative analysis on days 3, 5, and 7. By the stimulation of BMP9, ALP activities increased gradually from day 3 to day 7, which were dramatically higher than that of the AdGFP groups (Figure 2A,a,b). As the late osteogenic differentiation marker, Alizarin Red S staining was utilized on day 7 and 9, and we found extensive matrix mineralization with the stimulation of BMP9, clearly more than that of the AdGFP groups (Figure 2B). In addition, we found that mRNA expression levels of key osteogenic transcription factors Runt-related transcription factor 2 (Runx2) and Osterix were up-regulated by BMP9 (Figure 2C,a,b). mRNA expression levels of late osteogenic differentiation markers osteopontin (OPN) and bone sialoprotein (BSP) were also up-regulated with the stimulation of BMP9 (Figure 2C,c,d).

### BMP9-induced angiogenic differentiation of MSCs

**Figure 3:**
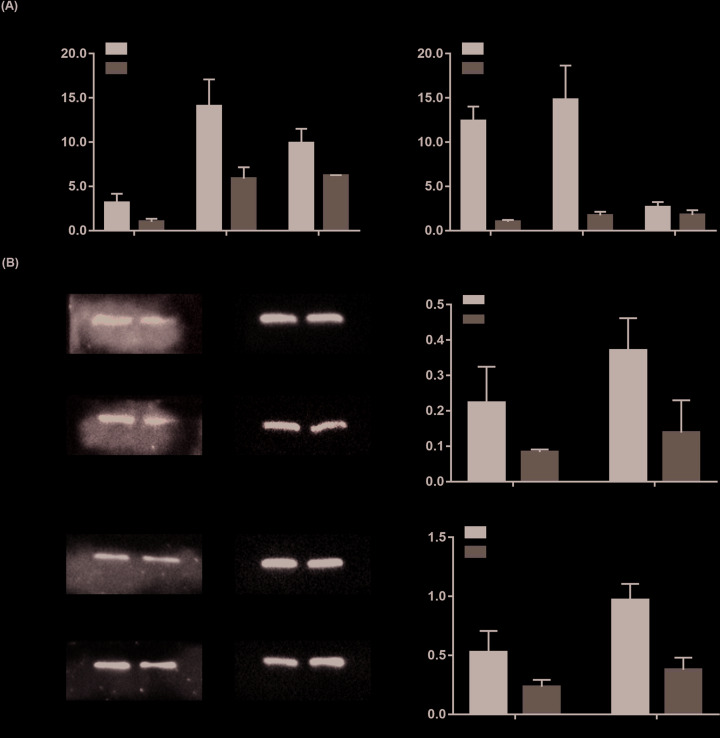
BMP9-induced angiogenic differentiation of MSCs *in vitro* (**A**) BMP9-induced angiogenic differentiation markers expression in MSCs at mRNA level. Subconfluent iMEF cells were infected with AdBMP9 or AdGFP. At the indicated time points, total RNA of each group was isolated and subjected to RT-qPCR analysis with VEGFa (**a**), CD31 (**b**) primers. All samples were normalized with the reference gene GAPDH. Relative expression was calculated by dividing the relative expression values (i.e., gene/GAPDH) in AdBMP9 group with that from the AdGFP group. Each assay condition was done in triplicate. ‘*’*P*<0.05, ‘**’*P*<0.01, AdBMP9 group vs. AdGFP group. (**B**) BMP9-induced angiogenic differentiation marker expression in MSCs at protein levels. Subconfluent iMEF cells were infected with AdBMP9 or AdGFP. At the indicated time points, Western blot was used for detecting the expression of VEGFa (**a**) and CD31 (**c**). Relative protein expression levels were analyzed by Image Lab software using GAPDH as control (**b,d**). The results are expressed as mean ± SD of triplicate experiments, ‘**’*P*<0.01, AdBMP9 group vs. AdGFP group.

**Figure 4:**
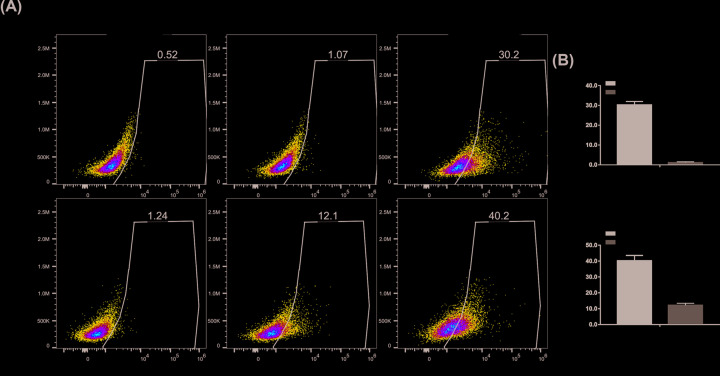
BMP9-induced CD31-positive cells analysis by flow cytometry (**A**) Subconfluent iMEF cells were infected with AdBMP9 or AdGFP. At the indicated time points, cells were resuspended and incubated with the CD31 antibody and subjected to flow cytometry analysis. Representative images are shown. (**B**) The total number of CD31-positive cells were quantified on day 3 (**a**) and day 5 (**b**), respectively. The results are expressed as mean ± SD of triplicate experiments, ‘**’*P*<0.01, AdBMP9 group vs. AdGFP group.

To further identify the angiogenic differentiation of iMEF cells with the stimulation of BMP9, we detected the expressions of VEGFa and endothelial cell (EC)-specific maker CD31 at both mRNA and protein levels. As shown in Figure 3A, BMP9 dramatically up-regulated VEGFa mRNA expression levels on day 3, then to the peak level on day 5, and finally decreased appropriately on day 7 (Figure 3A,a). As for the mRNA expression levels of CD31, the same trend was found (Figure 3A,b). Meanwhile, VEGFa and CD31 protein expression levels on days 3 and 5 were up-regulated by BMP9 through Western-blot testing (Figure 3B,a,c) and quantitative analysis, respectively (Figure 3B,b,d). In addition, flow cytometry was used to detect CD31-positive cells. As shown in Figure 4, with the induction of BMP9, the proportion of CD31-positive cells reached 30.2 and 40.2% on days 3 and 5, respectively, while the proportion of CD31-positive cells in the AdGFP group were 1.07 and 12.1% on days 3 and 5 (Figure 4A). The quantitative analysis showed the same trend (Figure 4B). These results indicate that BMP9 holds the potential to induce MSCs angiogenic differentiation. Interestingly, without the stimulation of BMP9, approximately 12.1% cells were identified as CD31-positive cells, which suggests the importance of further identifying the ability of BMP9-induced blood vessels formation *in vivo*.

### BMP9 exhibits dual and coupled roles in inducing MSCs osteogenic and angiogenic differentiation in the ectopic bone formation model

**Figure 5:**
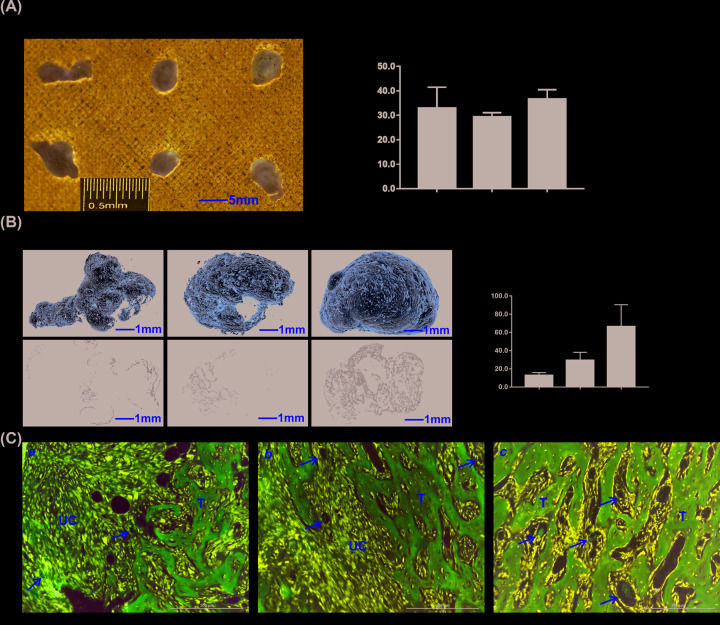
BMP9-induced osteogenesis and angiogenesis in the ectopic bone formation model (**A**) Macrographic images of ectopic masses. AdBMP9- or AdGFP-infected iMEF cells were implanted subcutaneously into the flanks of nude mice. Ectopic masses were retrieved at 3, 4, and 5 weeks (**a**), cells infected with AdGFP failed to form any discoverable masses. Volume of the masses at different time points were determined using Vernier calipers (**b**). (**B**) Micro-CT imaging (3D reconstructions and cross-sectional images) of the retrieved bone masses at different time points (**a**), scale bar = 1 mm. The micro-CT data were quantitatively analyzed to determine the average BV, BV/TV is presented (**b**). The results are expressed as mean ± SD, ‘**’*P*<0.01, compared with 3-week samples, ‘^##^’*P*<0.01, compared with 4-week samples. (**C**) Histological analysis of the retrieved samples. The retrieved samples were fixed, decalcified, paraffin-embedded, and subjected to H&E staining. Representative images are shown, scale bar = 200 μm, sinusoid capillary is indicated by arrows. Abbreviations: T, trabecular bone; UM, undifferentiated MSC.

Ectopic bone formation assays were conducted to explore BMP9-induced osteogenic and angiogenic differentiation of MSCs. Since BMP9-mediated bone formation is time-dependent, it was imperative to analyze BMP9-induced ectopic bone formation at different time points. With our previously constructed MSCs implantation assay, we injected iMEFs infected with AdGFP or AdBMP9 at the same infection ratio subcutaneously into the flanks of athymic nude (nu/nu) mice for 3, 4, and 5 weeks, respectively. The cells infected with AdGFP failed to form any discoverable mass. There was no macroscopic difference for the ectopic masses formed in different time points (Figure 5A,a). Meanwhile, the volume analysis exhibited no statistically significant difference among ectopic masses formed in different time points either (Figure 5A,b).

Next, the ectopic masses were subjected to μCT analysis. 3D images indicated robust bone formation with the induction of BMP9 and no observative differences were found among masses formed at 3, 4, and 5 weeks (Figure 5A,a). However, traverse images revealed that trabecular bone formed only in the edges of ectopic masses at 3 weeks, and then more trabecular bone increased from the edges to the center of the ectopic masses at 4 and 5 weeks (Figure 5B,a). In the quantitative analysis of trabecular bone with the utilization of BV/TV, we found that trabecular bone increased gradually from 3 to 5 weeks. These results indicate that the ectopic bone masses became more and more mature from 3 to 5 weeks (Figure 5B,b).

Histologic analysis of the retrieved bony samples was further carried out. At 3 weeks, only the edge of the ectopic masses formed trabecular bone, with abundant undifferentiated iMEF cells in the center, and sinusoid capillaries were observed in areas of undifferentiated iMEF cells (Figure 5C,a). At 4 weeks, more trabecular bone formed from edge to center and less undifferentiated iMEF cells were observed compared with the 3-week samples. Sinusoid capillaries were observed at both trabecular bone areas and undifferentiated iMEF cell areas (Figure 5Cb). At 5 weeks, a mass of trabecular bone formed with no discernible undifferentiated iMEF cells, and sinusoid capillaries were distributed among the trabecular bone (Figure 5C,c).

**Figure 6:**
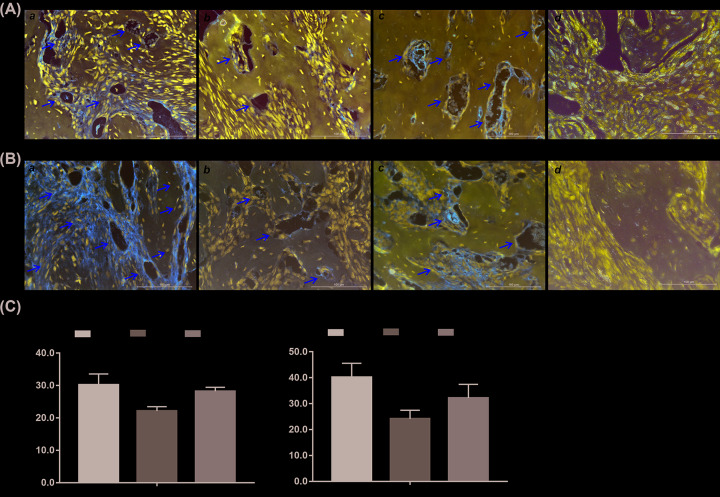
BMP9-induced angiogenic differentiation marker expression in the ectopic bone formation model (**A**) IHC staining of KDR expression in the retrieved ectopic bone masses at 3 weeks (**a**), 4 weeks (**b**), 5 weeks (**c**), and staining without primary antibody was used as negative control (**d**). The paraffin-embedded samples were sectioned, deparaffinized, and subjected to IHC staining using the KDR antibody. Species-specific IgG and minus primary antibody were used as negative controls (not shown). Representative images are shown, scale bar = 100 μm, positive staining is indicated by arrows. (**B**) IHC staining of CD31 expression in the retrieved ectopic bone masses at 3 weeks (**a**), 4 weeks (**b**), 5 weeks (**c**), and staining without primary antibody was used as negative control (**d**). The paraffin-embedded samples were sectioned, deparaffinized, and subjected to IHC staining using the CD31 antibody. Species-specific IgG and minus primary antibody were used as negative controls (not shown). Representative images are shown, scale bar = 100 μm, positive staining is indicated by arrows. (**C**) Semi-quantitative analysis of KDR positive cells (**a**) and CD31-positive cells (**b**). ‘**’*P*<0.01, 4 vs. 3 weeks, ‘^##^’*P*<0.01, 4 vs. 5 weeks.

Finally, we examined angiogenic differentiation marker expression in the retrieved bone masses through immunohistochemical staining (IHC staining). Endothelial growth factor receptor 2 (VEGFR-2), also known as KDR, is a specific marker of vasculogenesis was examined first. As shown in Figure 6A, KDR expression was detectable in the undifferentiated iMEF cells at 3 weeks, and then specifically expressed at walls of sinusoid capillaries which were distributed throughout the trabecular bone at approximately weeks 4–5. On the other hand, CD31 is a specific marker of ECs which stand for the process of angiogenesis. As shown in Figure 6B, an abundance of CD31-positive cells was detected among the undifferentiated iMEF cells and walls of sinusoid capillaries at 3 weeks, and then specifically expressed at walls of sinusoid capillaries in the trabecular bone at weeks 4 and 5. The quatitative analysis of KDR and CD31 positive cells are listed in Figure 6C. These results suggest that the angiogenic differentiation of MSCs is coupled with trabecular bone formation.

## Discussion

Effective bone tissue engineering holds the potential for the repair of large bone defects and nonunion fractures caused by trauma, regenerative diseases, tumor, or infection [1,2,4,38,39]. However, insufficient blood vessel formation may seriously limit the application of engineered bone [40–43]. Thus, effective osteogenesis and angiogenesis are essential for the construction of tissue engineered bone.

BMP9 is one of the most osteogenic BMPs that induce osteogenic differentiation of stem cells [12,13,15]. Several signaling pathways have been identified to help facilitate BMP9-induced osteogenic differentiation of MSCs [15,44]. Meanwhile, BMP9 was proved to regulate postnatal angiogenesis, which indicated the angiogenic differentiation potential of BMP9 [45,46]. In previous studies, we have identified that angiogenic signaling is essential for BMP9-mediated osteogenic differentiation of MSCs [16,30,31], however, BMP9-induced angiogenic differentiation of MSCs and the relationship between BMP9-mediated osteogenic and angiogenic differentiation of MSCs were not well established. In the present study, we found that BMP9 exhibited dual roles in inducing MSCs osteogenic and angiogenic differentiation *in vitro*, and further *in vivo* assays confirmed that BMP9-mediated bone formation are coupled with angiogenesis. Therefore, our results indicated that BMP9 induces both osteogenic and angiogenic differentiation of MSCs, suggesting that BMP9 is a potential growth factor for the construction of tissue engineered bone.

It is still controversial whether BMP9-induced angiogenic differentiation is earlier than trabecular bone formation [15,47]. In our *in vitro* tests, we found that BMP9 exhibited dual potential to induce MSCs osteogenic and angiogenic differentiation at early stage (day 3). The *in vivo* tests also supported that blood vessel formation markers expression were combined with trabecular bone formation. These results indicated that BMP9 simultaneously induced MSCs angiogenic and osteogenic differentiation rather than angiogenic differentiation posterior to osteogenic differentiation.

Recently, the blood vessel formation process was identified as two stages [48–50]. The first stage is vasculogenesis, when mesodermal precursor cells undergo angioblasts differentiation toward ECs. In this stage, VEGFR2, or KDR, is the earliest and specific marker. Fibroblast growth factor 2 (FGF2) and BMP4 were reported to initiate the specification of the mesoderm and differentiation toward ECs [48]. In the present study, we found that BMP9 up-regulates the expression of KDR at the early stage of osteogenesis in the ectopic bone formation model, which indicates that BMP9 could trigger the process of vasculogenesis. The second stage of the blood vessel formation process is the activation of ECs and the formation of new blood vessels, or called sprouting angiogenesis. In this stage, VEGFa stimulates the sprouting of ECs to form new vessels, and the markers of ECs were identified as the specific markers of this stage. Notch signaling, especially (Dll4)-Notch1 and Jagged1 (Jag1)-Notch1 interactions regulate this process [51–55]. Our previous studies demonstrated that the activation of Notch1 signaling promoted BMP9-mediated osteogenesis-angiogenesis coupling and further promoted bone formation [16]. In the present study, we identified that BMP9 up-regulates the expression of VEGFa and CD31 in mRNA and protein levels *in vitro*, which indicates that BMP9 holds the potential to induce MSCs differentiation into EC-like cells [56,57]. However, CD31-positive cells were also detected in the control group on day 5, which indicates the necessity of identifying the ability of BMP9-induced blood vessel formation *in vivo*. Furthermore, our *in vivo* assays confirmed that CD31-positive cells emerged in undifferentiated MSC areas, followed by sinusoid capillary formation. These results suggest that BMP9 mediates the process of angiogenesis.

Through the IHC staining and semi-quantitative analysis of IHC staining-positive cells, we found blood vessel formation marker expression earlier than trabecular bone formation at 3 weeks, and then further expression at the walls of sinusoid capillaries distributed among the trabecular bone at 4 weeks, and finally widely distributed expression at bone marrow-like areas (Figure 6). These results suggested that blood vessel formation promoted trabecular bone formation and bone maturation. Taken together, we infer that BMP9 holds the potential to induce MSCs angiogenic differentiation by promoting the processes of both vasculogenesis and angiogenesis.

As another member of BMP family, recombinant human BMP 2 (rhBMP-2) has been approved for clinical treatment of acute open tibial shaft fracture by the American Food and Drug Administration (FDA). However, ectopic ossification has been known to occur after treatment with BMP2 [58]. One of the reasons for ectopic ossification is deficient vascular ingrowth. Our previous studies found that BMP2 simultaneously induced osteogenic and chondrogenic differentiation of MSCs, and notably, BMP2-induced osteogenic differentiation appears as endochondral ossification [37,59]. In the present study, we found sinusoid capillary formation at the early stage of BMP9-induced bone formation. This indicates that, rather than endochondral ossification, BMP9-induced bone formation is coupled with vascular ingrowth at an early stage, and our analysis identified that BMP9-induced osteogenesis and angiogenesis are coupled. This may be another difference between BMP9 and other BMP family members.

It is reported that 3D scaffolds hold the potential to promote blood vessel formation and bone formation [60–62], although BMP9-mediated blood vessel formation was identified by both *in vitro* and *in vivo* tests, it is still necessary to investigate BMP9-mediated bone formation with the use of 3D scaffolds to further construct BMP9-mediated bone tissue engineering.

Since blood vessels carry oxygen and nutrients to all cells and remove waste products from tissues, angiogenesis is a prerequisite for osteogenesis [27,49,50,63]. It is accepted that osteogenesis and angiogenesis are coupled during the bone development [27,29]. Although BMP9-mediated osteogenesis-angiogenesis coupling was put forward in our previous studies, to the best of our understanding, that BMP9 exhibits dual and coupled roles in inducing osteogenic and angiogenic differentiation of MSCs was first shown in the present study. Mechanisms underlying BMP9-mediated angiogenic differentiation and osteogenesis-angiogenesis will be clarified in our further studies. On the other hand, additional research focused on bone defect repair and 3D scaffold-mediated bone formation need to be done to verify the potential of construction via BMP9-mediated bone tissue engineering.

## Conclusion

Taken together, these findings suggest that BMP9 exhibits dual and coupled roles in inducing osteogenic and angiogenic differentiation of MSCs. BMP9-induced osteogenesis is coupled with angiogenesis, thus BMP9 has potential for bone tissue engineering.

## Abbreviations

ALP: alkaline phosphatase
BMP: bone morphogenetic protein
BV: bone volume
EC: endothelial cell
HRP: horseradish peroxidase
IHC staining: immunohistochemical staining
iMEF: immortalized mouse embryonic fibroblast
KDR: kinase insert domain receptor
MSC: mesenchymal stem cell
PBS: phosphate buffer solution
PCR: polymerase chain reaction
PVDF: polyvinylidene fluoride
TGF-β: transforming growth factor β
TV: total volume
VEGFa: vascular endothelial growth factor a
3D: three-dimensional
μCT: micro-computed tomography
